# Dispersal of thermophilic beetles across the intercontinental Arctic forest belt during the early Eocene

**DOI:** 10.1038/s41598-017-13207-4

**Published:** 2017-10-11

**Authors:** Adam J. Brunke, Stylianos Chatzimanolis, Brian D. Metscher, Karin Wolf-Schwenninger, Alexey Solodovnikov

**Affiliations:** 1 Third Department of Zoology, Natural History Museum of Vienna, Burgring 7, 1010 Vienna, Austria; 2 Canadian National Collection of Insects, Arachnids and Nematodes, Agriculture and Agri-Food Canada, 960 Carling Avenue, Ottawa, K1A 0C6 ON Canada; 3 Department of Biology, Geology and Environmental Science, University of Tennessee at Chattanooga, 615 McCallie Ave, Dept. 2653, Chattanooga, TN 37403 USA; 4 Department of Theoretical Biology, University of Vienna, Althanstrasse 14, 1090 Vienna, Austria; 5 Staatliches Museum für Naturkunde Stuttgart, Rosenstein 1, 70191 Stuttgart, Germany; 6 Biosystematics, Natural History Museum of Denmark, Universitetsparken 15, 2100 Copenhagen, Denmark

## Abstract

Massive biotic change occurred during the Eocene as the climate shifted from warm and equable to seasonal and latitudinally stratified. Mild winter temperatures across Arctic intercontinental land bridges permitted dispersal of frost-intolerant groups until the Eocene-Oligocene boundary, while trans-Arctic dispersal in thermophilic groups may have been limited to the early Eocene, especially during short-lived hyperthermals. Some of these lineages are now disjunct between continents of the northern hemisphere. Although Eocene climate change may have been one of the most important drivers of these ancient patterns in modern animal and plant distributions, its particular events are rarely implicated or correlated with group-specific climatic requirements. Here we explored the climatic and geological drivers of a particularly striking Neotropical-Oriental disjunct distribution in the rove beetle *Bolitogyrus*, a suspected Eocene relict. We integrated evidence from Eocene fossils, distributional and climate data, paleoclimate, paleogeography, and phylogenetic divergence dating to show that intercontinental dispersal of *Bolitogyrus* ceased in the early Eocene, consistent with the termination of conditions required by thermophilic lineages. These results provide new insight into the poorly known and short-lived Arctic forest community of the Early Eocene and its surviving lineages.

## Introduction

Following the rise of angiosperm plants during the Cretaceous Terrestrial Revolution^1^, the most biologically significant event during the Cenozoic was arguably the transition of a widespread and equable ‘hothouse’ climate to a largely seasonal ‘icehouse’ climate’, varying strongly with latitude^2^. Just before this transition, mild winters during the Eocene and the presence of high-latitude land bridges allowed an Arctic rainforest and its associated fauna to extend across continents, up to 76–78°N paleolatitude^3,4^. This community, known widely as the ‘boreotropics’, was a unique mixture of frost-intolerant groups such as palms and crocodilians that now occur only in the paratropics and tropics, and frost-tolerant lineages such as the alders that today form elements of temperate forests^3,5,6^. Thermophilic lineages, those requiring high mean annual temperatures (MAT) in addition to mild winters, also formed these high-latitude communities during the early Eocene^7^, especially during three brief (170,000-2 Myr) hyperthermal periods (55.5-50.0 Mya)^8^, when forests warmed from upper microthermal/lower mesothermal^2–4^ to upper mesothermal/megathermal^9^.

As the climate polarized and the Arctic rainforests transitioned to temperate over the late Eocene and Oligocene^2^, the thermophilic and frost intolerant lineages went extinct over much or all of their Eocene distribution but some retreated toward the equator and survive today in single or multiple refugia, disjunct between continents of the northern hemisphere^10^. Refugial areas include the Neotropics and southern Nearctic, the Mediterranean, the Oriental region, and southern parts of the east Palearctic (e.g., Japan, Korea)^11^. Thus, Eocene climate change may have been one of the most important drivers of intercontinental disjunctions in Modern animal and plant distributions, along with the Cretaceous break-up of mega-continent Gondwana that created better-known disjunctions across the southern hemisphere.

Although Eocene divergences and origins within the boreotropics are commonly reported in phylogenetic studies, especially of plants and vertebrates^11,12^, particular climatic events during this long and complex transition are rarely implicated in triggering the divergence and even more rarely correlated with group-specific climatic requirements. This oversimplifies an understanding of macroecological change of communities over the Eocene. At least two major events should be recognized: an early Eocene end of dispersal opportunities for thermophilic groups^7,8^; and a late Eocene increase in seasonality and winter-severity, resulting in the disappearance of the frost-intolerant Arctic rainforest community^2^. Divergence time estimates from fossil-calibrated phylogenetic dating can provide important paleoclimatic context for intercontinental disjunctions. However, reliable estimates may be difficult to obtain due to extinction of the clades in question and difficulties with morphological clocks if only fossil taxa are available^13^. This is particularly problematic for the entirely extinct boreotropical formiciine ants^7^ and omomyid primates^14^. Thermophilic groups appear to have been heavily impacted by extinction and rarely are both New and Old World lineages extant.

**Figure 1:**
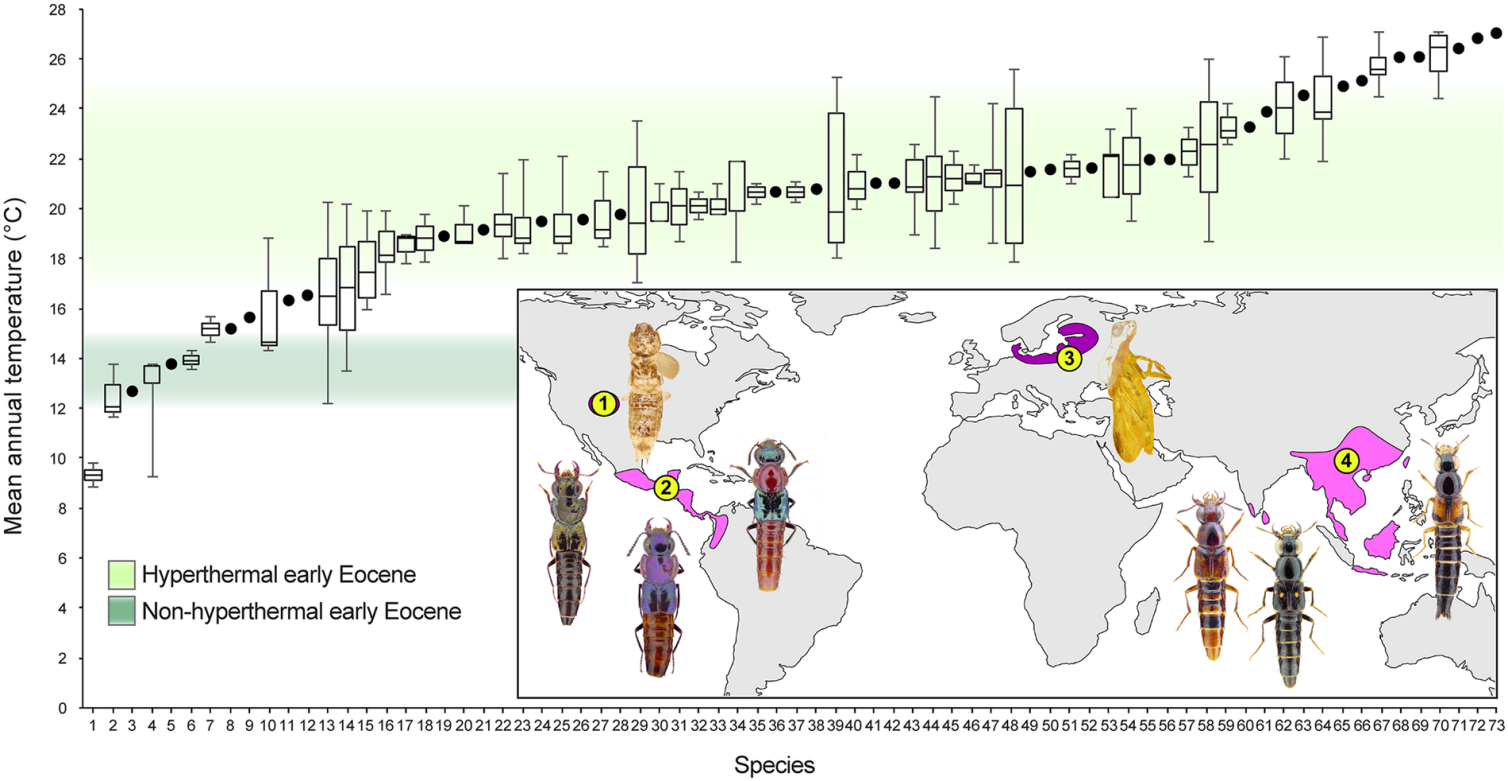
Mean annual temperature (MAT) for occurrences of *Bolitogyrus* species. Arctic hyperthermal and non-hyperthermal early Eocene MAT reconstructions from^2,4,9,32^. Single points indicate singular records and lines inside box plots correspond to median values. Inset: past and present global distribution of *Bolitogyrus*: 1, Green River, Colorado, North America (52 Mya); 2, extant Neotropical distribution; 3, Baltic region, Europe (33.9–37.8 Mya, possibly slightly older^19^); 4, extant Oriental distribution. Map created using QGIS 2.18^35^ and Adobe Illustrator CS6 (www.adobe.com).

The rove beetle *Bolitogyrus* Chevrolat is restricted to the Neotropical and Oriental regions and exhibits one of the most widely disjunct New-Old World distribution patterns in extant animals^15^. The genus is remarkable for its seventy-eight extant species, which are microhabitat specialists of fungus-covered deadwood^16^ (Fig. 1). Morphological and molecular evidence^15,17^ indicate single Neotropical and Oriental clades that are sister groups, suggesting a previously contiguous ancestral distribution split by a single event. *Bolitogyrus* belongs to a lineage of mostly northern hemisphere taxa within the hyperdiverse beetle tribe Staphylinini^17^. Staphylinini is thought to have originated in the Late Jurassic, though rigorous divergence estimates are unavailable for this group of more than 5,600 species^17^.

The above conditions together provide a rare opportunity to study the biogeographic origins of an Oriental-Neotropical disjunction in an extant animal group. Here we assess the role of major Eocene geological and climatological events in the intercontinental dispersal of and subsequent divergence within *Bolitogyrus* using a novel integration of evidence from Eocene fossils, a comprehensive occurrence dataset, paleoclimate reconstructions and fossil-calibrated divergence dating.

## Results

### Eocene paleodistribution of *Bolitogyrus*

**Figure 2:**
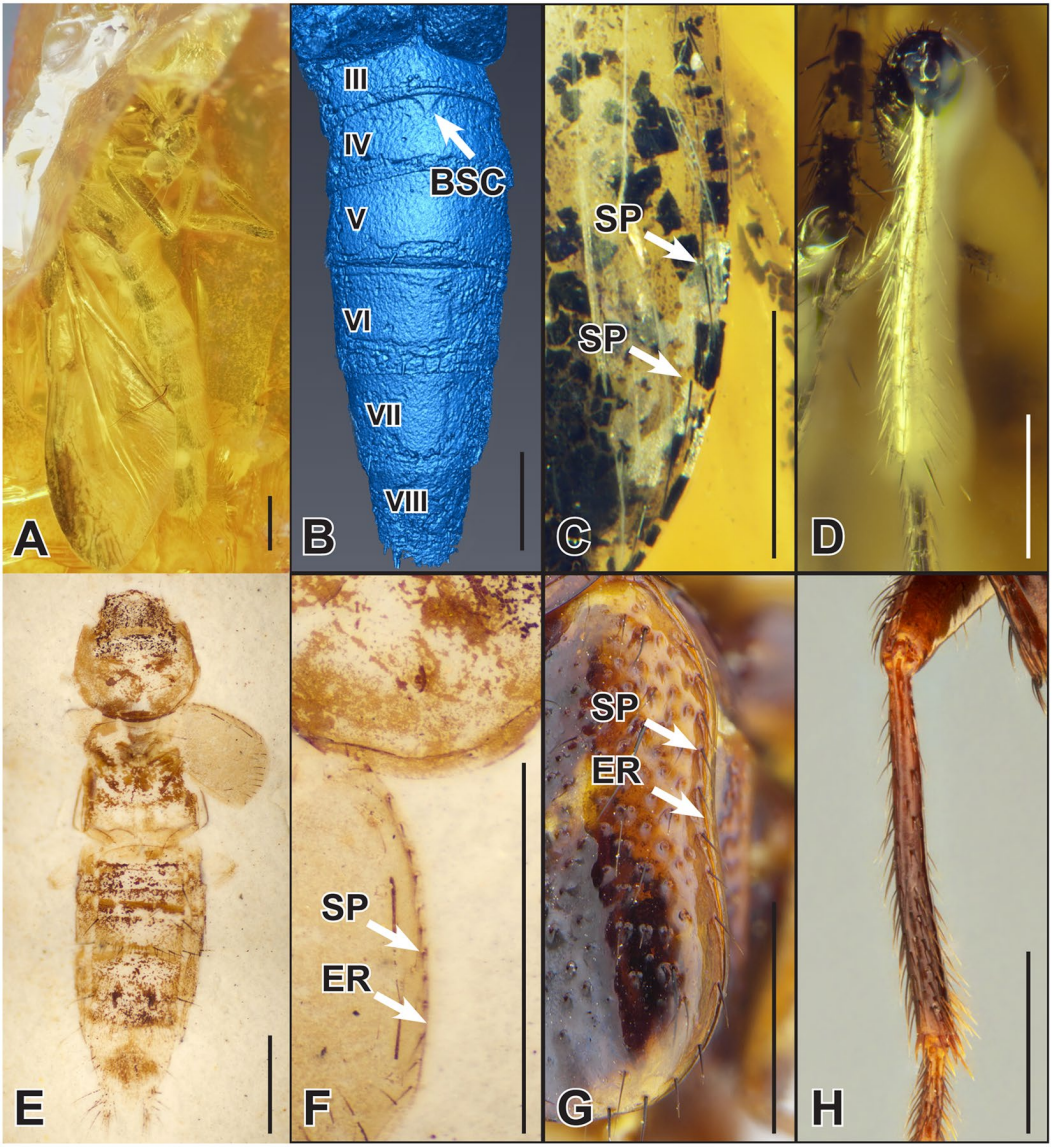
Fossil *Bolitogyrus*: Baltic amber (#BB-1937) (**A**–**D**), (**A**) lateral habitus, (**B**) microtomographic reconstruction of ventral abdomen, (**C**) elytral epipleuron in lateral view, (**D**) metatibia; Green River Formation (#PAL591468) (**E**–**F**), (**E**) dorsal habitus, (**F**) elytral epipleuron, images by D. Zyła (Denmark) and used with permission. Recent *Bolitogyrus*: (**G**) elytral epipleuron in lateral view, (**H**) metatibia. Scale bars: A, B, E, F, G = 1 mm; C, D, H = 0.5 mm. SP = setose puncture, ER = epipleural ridge, BSC = basal sternal carina.

Two Eocene fossils that could be unambiguously referred to *Bolitogyrus* were discovered in museum collections among hundreds of other staphylinid fossils (Fig. 2). Both specimens #BB-1937 (Baltic amber, ~33.9–37.8 Mya^18^, but see^19^ for an older estimate of ~44 Mya) (Fig. 2A) and #PAL591468 (Piceance Creek, Green River, 51.2 ± 0.52 Mya^20^) (Fig. 2E) possess a row of coarse macrosetal punctures along the elytral epipleuron (Fig. 2C,F,G) and can be confidently placed in the subtribe Cyrtoquediina (Staphylinini)^17^. In both fossils, this single epipleural row of punctures is situated near or in contact with a thickened epipleural ridge (Fig. 2C,F,G), a character state unique to *Bolitogyrus* ^16^. Additional character states visible in the Baltic amber specimen that are unique to *Bolitogyrus* within Cyrtoquediina include^16^: metatibia without spines (Fig. 2D,H) and abdominal sternite IV with basal sternal carina acutely projected at middle (Fig. 2B) (latter visible only in the X-ray micro tomography reconstruction).

### Divergence dates

Divergence estimates using lognormal fossil calibration priors (Analysis 1, see Methods) were remarkably similar (most 1–3% different, Table S2) to those using uniform fossil calibration priors (Analysis 2, see Methods). The divergence estimate for crown clade Quediina did not change (Table S2). Ninety-five percent highest probability density intervals for Analysis 2 were always much wider, and median values from Analysis 1 were about 1–4 Mya older than the former (Table S2). This suggests that our priors did not dominate the posterior estimates. In agreement with this, analyses run with ‘empty alignments’ always produced divergence estimates with strongly different posterior distributions compared to analyses run with data. As lognormal calibrations incorporate our prior knowledge of the staphylinid fossil record far better than the uniform calibrations (see Supplementary Information), we hereafter report the results of Analysis 1 unless otherwise stated.

**Figure 3:**
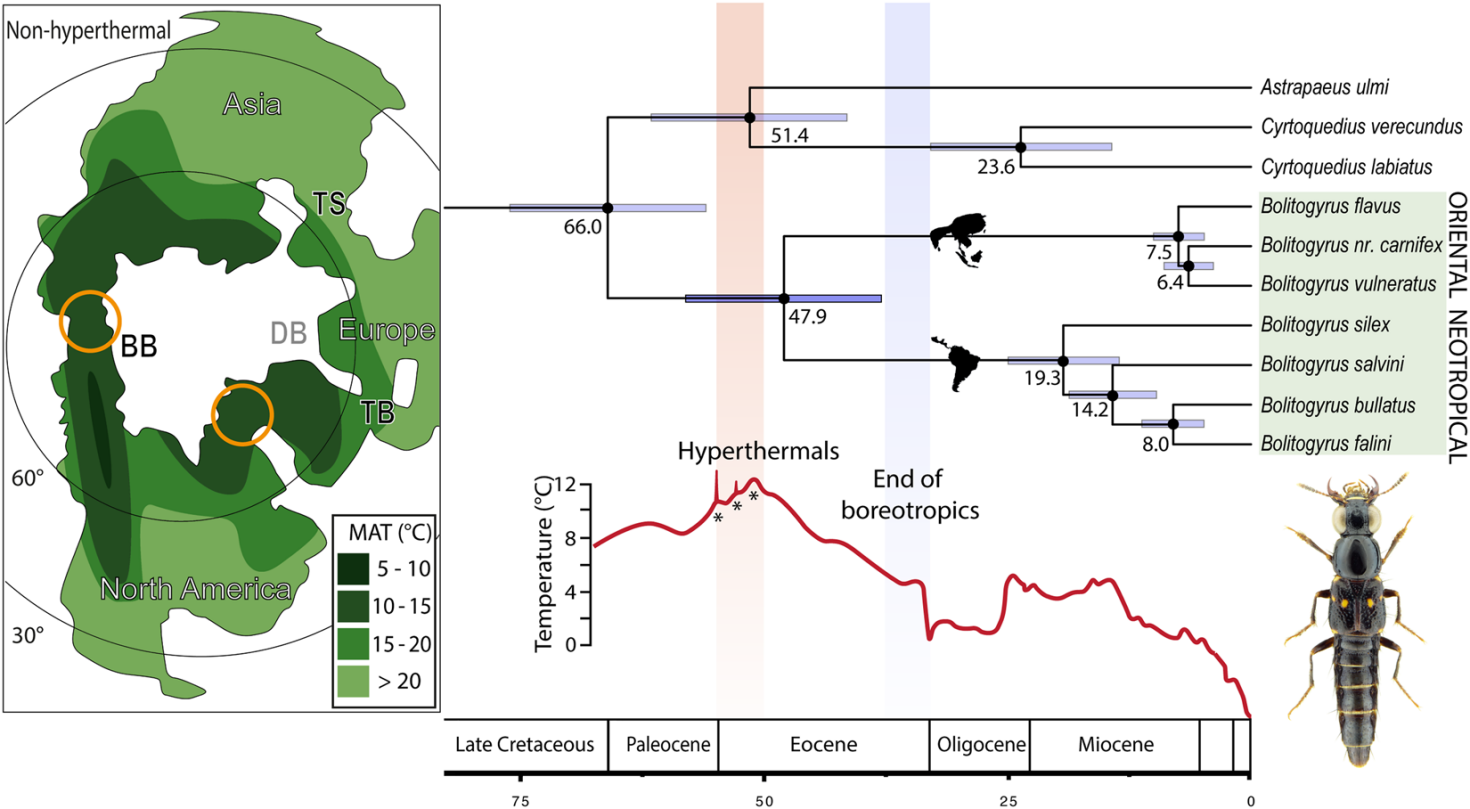
Divergence of New and Old World *Bolitogyrus* rove beetle lineages. Chronogram inferred by BEAST 1.8, with median ages in million years on their respective nodes and error bars representing 95% highest probability density for these estimates; for complete topology see Fig. 4. Global temperature curve over time estimated from oxygen isotopic patterns in deep-sea benthic foraminifera, which are proportional to temperature and total ice-sheet mass; re-drawn from^8^, copyright permission from The American Association for the Advancement of Science, license 4067140346587). Red bar represents dispersal of thermophilic organisms during early Eocene hyperthermals^9^ and blue bar represents decline of equable boreotropical community^2^. Paleoclimate and geological reconstructions for 55 Ma from^33^ (figures modified from^33^ and used here under CC BY 3.0 license: https://creativecommons.org/licenses/by/3.0/), orange circles mark high-latitude dispersal points, DB (grey) not emergent at this time^12^. BB = Bering Bridge; DB = DeGeer Bridge; TB = Thulean Bridge; TS = connections across Turgai Strait; MAT = mean annual temperature. Illustrations created using Adobe Illustrator CS6 (www.adobe.com).

**Figure 4:**
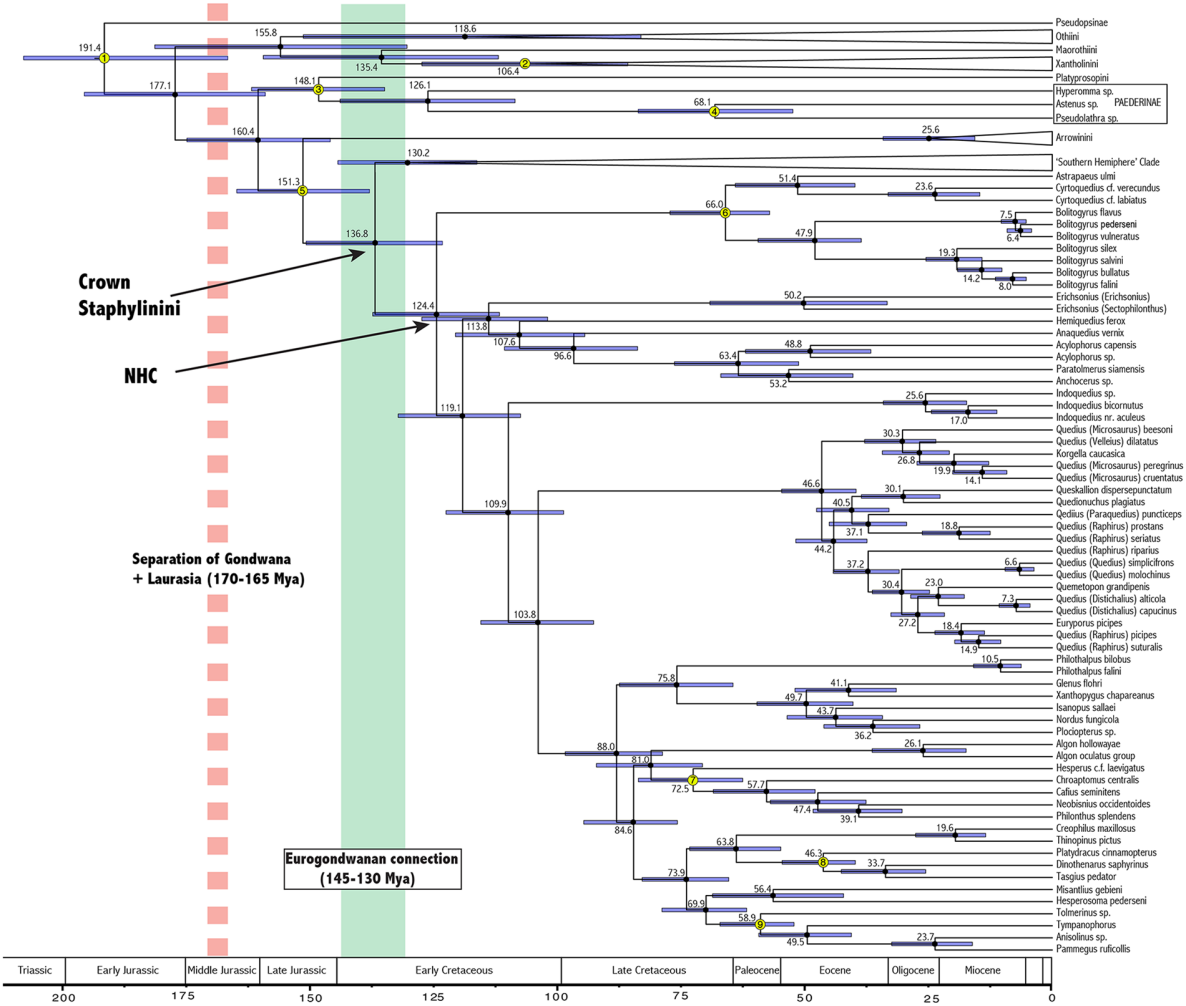
Chronogram of Staphylinini and outgroup taxa as inferred by BEAST 1.8, with median ages in million years on their respective nodes and error bars representing the 95% highest probability density for these estimates. Calibrated nodes are marked in yellow and correspond to numbers in Table S1. NHC – northern hemisphere clade of^17^. Broken red line indicates separation of Laurasia and Gondwana^21^ and green bar indicates a possible Eurogondwanan connection^36^.

We estimated an early Paleocene age (66.0, 57.1–77.2 Mya) for the ancestor of *Bolitogyrus* and an early Eocene age (47.9, 38.6–59.4 Mya) for the divergence of its Neotropical and Oriental lineages (Fig. 3). The complete chronogram for the tribe Staphylinini is given in Fig. 4 and a list of divergence estimates is given in Table S2.

### Climate profile of *Bolitogyrus*

More than ninety-one percent (67/73) of *Bolitogyrus* species regularly occur in forests with a Mean Annual Temperature (MAT) > 15 °C (Fig. 1). The majority of species occur in a narrow band of upper mesothermal to lower megathermal forests with MAT = 18–22 °C (Fig. 1). Exceptionally, six species occur in forests with MAT < 15 °C (*B*. *kitawakii* Smetana and Zheng, *B*. *nigropolitus* Smetana, *B*. *nigerrimus* Yuan *et al*., *B*. *electus* Smetana and Zheng, *B*. *strigifrons* (Wendeler) and *B*. *fukiensis* (Scheerpeltz)). Except for *B*. *strigifrons* (Coldest Month Mean Temperature (CMMT) = 11 °C), these same species tolerate CMMT < 5 °C. The remaining species are restricted to forests with CMMT > 5 °C and the vast majority (>91%) with CMMT > 8 °C. The northernmost species of the genus, Chinese *Bolitogyrus kitawakii*, uniquely occurs in forests with CMMT < 0 (−1.0–−0.7). MAT values for *Bolitogyrus* ranged from 8.9–27.1 °C and CMMT values ranged from −1.0–26.7 °C. Extant and paleodistributions of the genus are shown in Fig. 1 (inset).

## Discussion

As indicated by fossils (Fig. 2), the Eocene stem distribution of *Bolitogyrus* was very different than today and included North America and Europe, where the genus no longer occurs. We consider these fossils as stem group as both taxa must have diverged from the crown lineage after the acquisition of most derived traits of *Bolitogyrus* but before the evolution of the elytral punctation characteristic of extant species. Our early Paleocene age estimate for the stem origin of this lineage greatly post-dates the Jurassic break-up of Laurasia^21^ (Fig. 4) and implies high latitude inter-continental dispersal across Arctic land bridges (Fig. 3). Extinction of intermediates has made it impossible to ascertain the area of origin or dispersal direction for stem *Bolitogyrus*, as the extant diversity is contained within completely allopatric sister clades, and the sister group to the genus consists of another allopatric pair of sister clades (Palearctic-Neotropical) (Fig. 3). Divergence time estimates for major lineages of Staphylinini (Fig. 4) and their potential biogeographic implications are discussed separately in the Supplementary Information.

Based on the divergence time estimate for its Neotropical and Oriental clades, intercontinental dispersal of *Bolitogyrus* ceased during the early Eocene, far before the end of mild winters (CMMT > 5 °C)^2^ on Arctic land bridges during the late Eocene^2,12^. The divergence in *Bolitogyrus* was instead coincident with the termination of maximal MAT, highest during three brief hyperthermal periods^7^ (Fig. 3), at the end of the early Eocene. This suggests that the ancestor of Neotropical and Oriental clades was a thermophile requiring both high MAT and mild winters.

Over ninety-one percent of extant *Bolitogyrus* species require humid, ever-wet forests with MAT > 15 °C and CMMT > 8 °C. The few extant *Bolitogyrus* adapted to cooler, high-elevation forests with colder winter temperatures, better characterized as frost-intolerant rather than thermophilic, are scattered across Neotropical and Oriental clades in three different species groups^15,16^ that also include upper-mesothermal or megathermal forest species. These frost-intolerant exceptions are here interpreted as recent adaptations of the lineage to high elevation, warm temperate forests, derived from lower-elevation, thermophilic ancestors. This interpretation is consistent with our divergence estimate and is far more parsimonious than numerous, independent adaptations to upper-mesothermal and megathermal environments. After the final hyperthermal period in the latest early Eocene^8^, the climate on forested Arctic land bridges returned to cooler MAT values, outside of the climate profiles of over 90% of extant *Bolitogyrus* species. We propose that the early Eocene divergence of *Bolitogyrus* into two disjunct lineages was driven by changes in MAT, ending intercontinental dispersal that mostly likely occurred during one of the early Eocene hyperthermals. Although humidity is another important climatic factor for *Bolitogyrus*, precipitation remained high into the Oligocene^2,4^ and is not likely to have played a role in its divergence into two major lineages. Neotropical and Oriental *Bolitogyrus* species share the same association with humid, fungus-covered deadwood microhabitats^15,16^, and so it is also parsimonious to conclude that this specialization already evolved in stem *Bolitogyrus* at least 50 Mya ago.

Based mostly on evidence from the fossil record, early Eocene hyperthermals were a time of rapid biological exchange between continents. The first of these hyperthermals, the Paleocene-Eocene Thermal Maximum, permitted intercontinental dispersal in newly emerged mammal lineages including primates, and the even and odd-toed ungulates^14,22^. An early Eocene divergence estimate (46.7, 42.4–50.8 Mya) for the separation of primates into New and Old World lineages^23^ suggests that intercontinental dispersal in this primarily thermophilic group may have been driven by hyperthermals. Giant ants of the extinct subfamily Formiciinae are known only from megathermal Eocene Europe and North America^7^. Based on their body size, they were even more thermophilic than *Bolitogyrus* and Arctic dispersal was certainly constrained to hyperthermal events^7^. Although modern tapirs are restricted to megathermal environments in the Neotropical and Oriental regions, similar to *Bolitogyrus*, they did not evolve until the Oligocene^24^ and must have dispersed across Arctic land bridges under more seasonal conditions. To our knowledge, very few examples of thermophilic Eocene relicts remain today, though we expect more examples to come to light among the diverse insects.

Far more frequently, at least as reported in the literature, extant boreotropical relicts show divergences into New and Old World lineages before (Paleocene^25^) and after (late Eocene^26–28^) the early Eocene, and their distributions are probably constrained by mild winter temperatures, as in the palms^29^. At the Eocene-Oligocene boundary, winter temperatures decreased, were more different from summer temperatures and became less constant between years^2^, coincident with New-Old World divergences in swallowtail butterflies^26^, tropical ferns^28^ and the plums (*Prunus*)^27^; the former dispersing southward at this time, possibly with frost-intolerant host plants that were gradually replaced by a more temperate flora. A similar situation likely occurred with palm-associated seed-beetles^29^, whose potential hosts are known from Arctic pollen fossils until about the late Eocene^2^ but divergence estimates are unavailable. A broad comparative study of Eocene relicts, their divergences and climatic profiles may provide a community-level perspective on macro-ecological change through the Eocene.

Using evidence from the fossil record and divergence dating, we infer an intercontinental Eocene paleodistribution for stem *Bolitogyrus* and reconstruct a divergence estimate of its New and Old World crown lineages coincident with the end of Arctic dispersal opportunities for thermophilic organisms during the early Eocene. Based on climatic constraints exhibited by over 90% of its extant species, the ancestor of New and Old world *Bolitogyrus* most likely relied on short, hyperthermal elevations of MAT for intercontinental dispersal. A comprehensive phylogeny of this rarely collected genus, not yet available, may further help with determining the ancestral MAT limits of *Bolitogyrus*. *Bolitogyrus* is remarkable among thermophilic Eocene relicts for avoiding widespread extinction. Perhaps adaptations to a broader range of mesothermal and megathermal conditions allowed it to persist as a moderately diverse group, unlike Eocene megathermal specialists or those herbivorous on megathermal vegetation. An intercontinental Eocene paleodistribution in *Bolitogyrus*, a specialist of humid microhabitats within fungus covered deadwood, provides further evidence for a continuous Arctic belt of forest, as suggested by^30^. Even more remarkable is that *Bolitogyrus* has maintained its microhabitat specializations for at least 50 Mya and despite this, has survived the greatest global macroevolutionary change of the Cenozoic.

## Methods

### Morphological study

Fossil specimens of *Bolitogyrus* are deposited in the Staatliches Museum für Naturkunde Stuttgart (Stuttgart, Germany) (#BB-1937) and the National Museum of Natural History (Washington D.C., United States of America) (#PAL591468). Fossils were studied using a Leica MZ APO stereomicroscope. Specimen #PAL591468 was photographed using a Visionary Digital photomontage system under 70% alcohol to view fine details. MicroCT imaging of specimen #BB-1937 was performed at 4X magnification using an Xradia MicroXCT-200 system and tomographic sections were reconstructed with an isotropic voxel size of 5.1 μm. Segmentation and volume rendering were accomplished in Amira 6.0.1. The dataset was deposited in the University of Vienna’s Phaidra archive and is available here: https://phaidra.univie.ac.at/view/o:448456. Photomontage was accomplished using a motorized Nikon SMZ25 microscope and NIS Elements BR v4.5. Photos were processed in Adobe Photoshop CS6. Illustrations and maps were created using Adobe Illustrator CS6.

### Divergence dating

Divergence dating was performed in BEAST v1.8^31^ using a molecular dataset (six genes, 4730 bp) broadly encompassing Staphylinini diversity and several outgroup tribes^17^, and evidence from nine rove beetle fossil calibrations (Supplementary Tables S1 and S3). Further *Bolitogyrus* species were sequenced *de novo* and included as available (*B*. *pederseni* Brunke, *B*. *falini* Brunke, *B*. *silex* Brunke). Fossil calibration priors were given lognormal (Analysis 1) or uniform distributions (Analysis 2) (see Supplementary Methods). All divergence estimates are given as medians with their 95% high probability density in brackets. The dating analyses are further detailed in the Supplementary Information.

### Climate and occurrence data

MAT categories for vegetation (e.g., mesothermal) follow^7^: microthermal < 13 °C, mesothermal 14–19 °C inclusive, megathermal > 20 °C. Here we use the use the contrasting terms thermophilic and frost-intolerant to distinguish between organisms which require high MAT and mild winters, and mild winters only, respectively. MAT and CMMT estimates for non-hyperthermal early Eocene Arctic follow those of ^2–4,32^, which, given error margins, broadly agree on MAT = 12–15 °C and CMMT > 5 °C. Analyses suggest 5−10 °C warmer MAT and a CMMT > 8 °C for hyperthermal early Eocene Arctic^9^. Late Eocene (>38 Mya) values of MAT = 10–11 °C and CMMT < 5 °C follow^2^. A more detailed visualization of non-hyperthermal early Eocene (55 Mya) vegetation was approximated using climate reconstruction maps^33^.

An occurrence record dataset for all known specimens of *Bolitogyrus* was compiled using published primary data by the first author^15,16^. Duplicate localities and non-georeferenced records were removed to produce a dataset representing all Neotropical and all but five of fifty Oriental species. To create a climate profile for the genus (similar to^4^), current MAT and winter temperature (CQMT = coldest quarter mean temperature, used as proxy to CMMT as in^29^) data were derived from WorldClim v1.4^34^ at 30 s resolution and associated with occurrence records in QGIS v2.14 using the Point Sampling plugin by B. Jurgiel. Outliers were identified as values 2 °C below 10^th^ and above 90^th^ percentiles, and removed only for those species with 5 or greater records. Overall, 1 MAT outlier each was removed for 2 species, 1 CMMT outlier removed for 1 species, and 3 MAT outliers were removed for *B*. *costaricensis* (Wendeler), one of the most commonly collected species. The final dataset of 277 records is included in Supplementary Dataset 1.

### Data Availability

Novel DNA sequences can be accessed under GenBank accession numbers MF621983-MF621999. GenBank accession numbers of all sequences are given in the Supplementary Information.

## Electronic supplementary material

Supplementary information

Supplementary Dataset 1

## References

[1] Lloyd GT (2008). Dinosaurs and the Cretaceous TerrestrialRevolution. Proceedings of the Royal Society of London B: Biological Sciences.

[2] Eldrett JS, Greenwood DR, Harding IC, Huber M (2009). Increased seasonality through the Eocene to Oligocene transition in northern high latitudes. Nature.

[3] Basinger, J. F., Greenwood, D. R. & Sweda, T. In *Cenozoic plants and climates of the Arctic* Vol. 127 (eds M.C. Boulter & H.C. Fisher) 175-198 (Springer-Verlag, 1994).

[4] Greenwood DR, Basinger JF, Smith RY (2010). How wet was the Arctic Eocene rain forest? Estimates of precipitation from Paleogene Arctic macrofloras. Geology.

[5] Estes R, Hutchison JH (1980). Eocene lower vertebrates from Ellesmere Island, Canadian arctic archipelago. Palaeogeography, Palaeoclimatology, Palaeoecology.

[6] Archibald SB, Farrell BD (2003). Wheeler’s dilemma. Acta Zoologica Cracoviensia.

[7] Archibald SB, Johnson KR, Mathewes RW, Greenwood DR (2011). Intercontinental dispersal of giant thermophilic ants across the Arctic during early Eocene hyperthermals. Proceedings of the Royal Society B.

[8] Zachos J, Pagani M, Sloan L, Thomas E, Billups K (2001). Trends, rhythms, and aberrations in global climate 65 Ma to present. Science.

[9] Sluijs A (2009). Warm and wet conditions in the Arctic region during Eocene Thermal Maximum 2. Nature Geoscience.

[10] Morley, R. J. *Cretaceous and Tertiary Climate Change and the Past Distribution of Megathermal Rainforests*. 1–31 (Praxis Publishing, 2007).

[11] Sanmartín I, Engoff H, Ronquist F (2001). Patterns of animal dispersal, vicariance and diversification in the Holarctic. Biological Journal of the Linnean Society.

[12] Brikiatis L (2014). The De Geer, Thulean and Beringia routes: key concepts for understanding early Cenozoic biogeography. Journal of Biogeography.

[13] Lee, M. S. Y. Multiple morphological clocks and total-evidence tip-dating in mammals. *Biology Letters* **12**, 10.1098/rsbl/2016.0033 (2016). 10.1098/rsbl.2016.0033 PMC4971162 27381882

[14] Beard K (2008). The oldest North American primate and mammalian biogeography during the Paleocene–Eocene Thermal Maximum. Proceedings of the National Academy of Sciences.

[15] Brunke A, Solodovnikov A (2014). A revision of the Neotropical species of *Bolitogyrus* Chevrolat, a geographically disjunct lineage of Staphylinini (Coleoptera, Staphylinidae). ZooKeys.

[16] Brunke, A. A revision of the Oriental species of *Bolitogyrus* Chevrolat (Coleoptera: Staphylinidae). *ZooKeys*, 98 pp (Accepted). 10.3897/zookeys.664.11881 PMC5523158 28769623

[17] Brunke AJ, Chatzimanolis S, Schillhammer H, Solodovnikov A (2016). Early evolution of the hyperdiverse rove beetle tribe Staphylinini (Coleoptera: Staphylinidae: Staphylininae) and a revision of its higher classification. Cladistics.

[18] Perkovsky EE, Rasnitsyn AP, Vlaskin AP, Taraschuk MV (2007). A comparative analysis of the Baltic and Rovno amber arthropod faunas: representative samples. African Invertebrates.

[19] Ritzkowski S (1997). K-Ar-Altersbestimmungen der bernsteinführenden Sedimente des Sam- landes (Paläogen, Bezirk Kaliningrad). Metalla, Bochum.

[20] Smith ME, Carroll AR, Singer BS (2008). Synoptic reconstruction of a major ancient lake system: Eocene Green River Formation, western United States. Geological Survey of America Bulletin.

[21] Scotese, C. R. *Middle Jurassic paleographic map*. 295 (Geological Society of America Special Paper 288, 1994).

[22] Gingerich PD (2006). Environment and evolution through the Paleocene–Eocene thermal maximum. Trends in Ecology and Evolution.

[23] Pozzi L (2014). Primate phylogenetic relationships and divergence dates inferred from complete mitochondrial genomes. Molecular Phylogenetics and Evolution.

[24] Ashley MV, Norman JE, Stross L (1996). Phylogenetic analysis of the Perissodactylan family Tapiridae using mitochondrial cytochrome c oxidase (COII) sequences. Journal of Mammalian Evolution.

[25] Praz CJ, Packer L (2014). Phylogenetic position of the bee genera Ancyla and Tarsalia (Hymenoptera: Apidae): A remarkable base compositional bias and an early Paleogene geodispersal from North America to the Old World. Molecular Biology and Evolution.

[26] Condamine FL, Sperling F, Wahlberg N, Rasplus J-Y, Kergoat GJ (2012). What causes latitudinal gradients in species diversity? Evolutionary processes and ecological constraints on swallowtail biodiversity. Ecology Letters.

[27] Chin S-W, Shaw J, Haberle R, Wen J, Potter D (2014). Diversification of almonds, peaches, plums and cherries – Molecular systematics and biogeographic history of *Prunus*. Molecular Phylogenetics and Evolution.

[28] Wei R (2015). Eurasian origin, boreotropical migration and transoceanic dispersal in the pantropical fern genus *Diplazium* (Athyriaceae). Journal of Biogeography.

[29] Archibald SB, Morse GE, Greenwood DR, Mathewes RW (2014). Fossil palm beetles refine upland winter temperatures in the Early Eocene Climatic Optimum. Proceedings of the National Academy of Sciences.

[30] Smith T, Rose KD, Gingerich PD (2006). Rapid Asia–Europe–North America geographic dispersal of earliest Eocene primate *Teilhardina* during the Paleocene–Eocene Thermal Maximum. Proceedings of the National Academy of Sciences.

[31] Drummond A, Suchard M, Xie D, Rambaut A (2012). Bayesian phylogenetics with BEAUti and the BEAST 1.7. Molecular Biology and Evolution.

[32] Eberle JJ (2010). Seasonal variability in Arctic temperatures during early Eocene time. Earth and Planetary Science Letters.

[33] Huber M, Caballero R (2011). The early Eocene equable climate problem revisited. Climate of the Past.

[34] Hijmans RJ, Cameron SE, Parra JL, Jones PG, Jarvis A (2005). Very high resolution interpolated climate surfaces for global land areas. International Journal of Climatology.

[35] QGIS Development Team. QGIS Geographic Information Sytstem. *Open Source Geospatial Foundation*. http://qgis.osgeo.org (2009)

[36] Ezcurra MD, Agnolín FL (2012). A New Global Palaeobiogeographical Model for the Late Mesozoic and Early Tertiary. Systematic Biology.

