# Supplementary material for: Dispersal of thermophilic beetles across the intercontinental Arctic forest belt during the early Eocene
Source: Sci Rep. 2017 Oct 11;7:12972. doi: 10.1038/s41598-017-13207-4 (PMC5636899; doi:10.1038/s41598-017-13207-4)
Supplement: Supplementary file 1 — Supplementary information [file 41598_2017_13207_MOESM1_ESM.pdf]

Supplementary Information for:

Dispersal of thermophilic beetles across the intercontinental Arctic forest belt during the early Eocene

Adam J. Brunke, Stylianos Chatzimanolis, Brian D. Metscher, Karin Wolf-Schwenninger and Alexey Solodovnikov

Methods

Institutional abbreviations are as follows: *CNUB*, Key Laboratory of Insect Evolution and Environmental Changes, Capital Normal University, Beijing, China; *ISEA*, Institute for Systematics and Evolution of Animals, Polish Academy of Sciences, Kraków, Poland; *MCZ*, Museum of Comparative Zoology, Harvard University, Massachusetts, United States; *NMNH*, National Museum of Natural History, Smithsonian Institution, Washington D.C., United States; *SIZK*, Schmalhauzen Institute of Zoology, National Academic of Science, Kiev, Ukraine; *UTCI*, University of Tennessee at Chattanooga, Tennessee, United States; *VMNH*, Virginia Museum of Natural History, Virginia, United States; *YPM*, Peabody Museum of Natural History, Yale University, Connecticut, United States; *ZMUC*, Zoological Museum, University of Copenhagen, Copenhagen, Denmark.

Molecular dataset

A molecular dataset, originally created for phylogeny reconstruction and reclassification of the rove beetle tribe Staphylinini (Staphylininae)[1], was used here (with some modifications given below) for divergence dating as it includes *Bolitogyrus*, the northern hemisphere clade of Staphylinini and a wide range of outgroups that maximizes the available fossil calibrations. Three additional species of *Bolitogyrus* (*B. silex* Brunke, *B. falini* Brunke and *B. pederseni* Brunke) were sequenced *de novo* and added to the present dataset (see[1] for laboratory and vouchering protocols; Table S3 for voucher and Genbank data). Three outgroup taxa that were recovered on long branches[1] were pruned from the present dataset to prevent potential dating artefacts: Oxyporinae (*Oxyporus* Fabricius), the farthest outgroup taxon and Diochini (two *Diochus* Erichson species), a tribe distant from Staphylinini that unexpectedly appeared outside of the xantholinine lineage where it is typically considered to belong[2,3]. This modified dataset consisted of 4730 bp from six gene fragments (all but one nuclear) and 90 taxa. The dataset was re-aligned, edited and partitioned following procedures in[1].

Divergence dating

All divergence dating analyses were performed in BEAST 1.8.0[4] running on the CIPRES Science Gateway v3.3. (phylo.org). Each analysis consisted of two independent runs of at least 50 million generations, with trees sampled every 5000 generations, and their convergence was visualized in Tracer v1.6[5], ensuring all ESS values were well above 200. A guide tree was provided to BEAST based on the best ML tree of[1], as this topology did not contain polytomies. Tree topology was initially estimated simultaneously with node heights because the dataset used in this study differed slightly from that of[1]. As the resulting topology did not change (not shown here) with respect to the well supported nodes of[1], topology was fixed to save computational time in the subsequent analyses. Topology was linked between partitions but the clock and site partitions were each unlinked. For all analyses, an uncorrelated lognormal relaxed clock was used with a birth-death tree prior, since what little

of the Staphylinini fossil record is known suggests high levels of extinction, violating the assumptions of the simpler Yule prior, which models only speciation ('birth')[4]. Most priors were left at their default values but ucld.mean and ucld.standarddeviation were set to a mean of 0.1, with an exponential distribution (as recommended by N. Wahlberg (*pers. comm.*). Post-burn in trees were combined using LogCombiner and maximum clade credibility trees were created using TreeAnnotator, with additional annotation in FigTree v1.4.2 (http://tree.bio.ed.ac.uk/software/figtree) and Adobe Illustrator CS6. Analyses were also run with 'empty' alignments to examine the interaction effects of priors and to test for overly strong priors.

Fossil calibrations were constructed following the recommendations of the literature with respect to prior distributions[6], with respect to fossil documentation, phylogenetic assignment and date justification[7] (see Table S1). Only the oldest fossils for a given node were used in calibrations and the youngest age estimates of fossil deposits from the literature were used as hard minimum bounds (Table S1). All divergence age estimates are given in the text as medians with their 95% HPD in brackets.

Analysis 1

We used calibration priors similar to[8], with lognormal distributions and means that reflected a reasonable expected lag-time between the fossil and the true most recent common ancestor (MRCA) of a clade. While we acknowledge that any expected lag time is somewhat subjective, the means chosen for the nodes calibrated with Early Cretaceous (124–126 Mya) fossils of the Chinese Yixian formation (Table S1) require additional justification. Three higher taxa included in the present analysis have their oldest known representatives from this fossil locality: Arrowinini, Staphylinini (both Staphylininae) and Paederinae. However, relatively few staphylinid containing fossil deposits older than the Early Cretaceous are known, which leaves the possibility open that 124 Mya is a gross underestimate of their stem ages. Further, all three were diverse by the Early Cretaceous, implying a much older age and all have lineages asymmetrically distributed in both Laurasian and Gondwanan landmasses[1,2,9]. This suggests that stem groups of Arrowinini, Staphylinini and Paederinae were present on Pangaea sometime before Gondwana severed its last direct connection with a Laurasian landmass, namely North America[10]. This final separation developed over the Oxfordian and Callovian Periods of the Late Jurassic (160–164.7 Mya)[10,11,12], though direct, broad land connections were present as late as 170 Mya[13]. Therefore, the mean in real space of the lognormal distribution for these calibrations was set to 52 so that the median of these prior distributions was 170 Mya, a time when direct connections still existed. This parameter value placed high prior probability around 170 Mya but also allowed for much older or younger posterior age estimates. All other calibrated nodes were given a similar prior distribution except that the mean, and expected lag time, was 20 rather than 52, as all fossils were considered to post-date the origin of their stem taxon (usually multiple taxa known from the deposit) but much less so than the Yixian formation. For standard errors, we preferred the smaller (less strict) values (0.5) of those used by[8] as this allowed for a broader range of lag times between fossil and ancestor with high prior probability density (visualized as a more 'rounded top' of the distribution curve).

Analysis 2

A recent study on beetle evolution[14] opted to place relatively uninformative uniform priors on calibrated nodes with hard minimum bounds instead of modeling the prior distribution with an exponential or lognormal distribution. The potential benefit of this strategy may be the elimination of the subjectivity introduced when designating the mean and/or standard error of these distributions. To examine the effect of placing less informative

priors (uniform) with fewer parameters (only minimum and maximum bounds), we also ran an analysis (Analysis 2) with uniform calibrations featuring hard minimum bounds (values as in Analysis 1).

Fossil evidence for calibrations

A total of nine calibrations were placed on nodes (Table S1), including a prior constraint on the root with a uniform distribution and hard maximum bound of 220 Mya based on the oldest known staphylinid fossil *Leehermania prorova* Chatzimanolis et al. from the Cow Branch formation of Virginia[15]. Calibration 2 was placed on the node representing crown group Xantholinini based on two undescribed taxa in Baltic amber belonging to two very different genera. We herein report the tribe Xantholinini from Baltic amber for the first time. The calibration was placed on the crown node in this case as both specimens (Fig. S1A–B) are similar in general body form, and the punctation of the head and pronotum to the diverse 'small-bodied' xantholinine genera that form a clade (represented by *Neohypnus* Coiffait and Saiz, *Lithocharodes* Sharp*, Nudobius* C.G. Thomson) sister to the genus *Thyreocephalus* Guérin Méneville[1]. Calibration 3 was placed on the node leading to the stem of Paederinae based on several paederine taxa described from the Early Cretaceous Yixian Formation of China and placed in the extinct genus *Mesostaphylinus* Zhang[2]. The calibration was placed at the stem as these taxa are not yet confidently identified as any major extant lineage of Paederinae. Calibration 4 was placed on the node representing the most recent common ancestor shared between the Lathrobiina (here *Pseudolathra* Casey) and the Asteniina (here *Astenus* Dejean) of Paederinae, based on the presence of diverse, multiple fossil *Lathrobium* Gravenhorst species from Baltic amber[16].

Calibration 5 was placed on the node leading to the stem of Staphylinini based on several species of the extinct genus *Cretoquedius* Ryvkin from the Yixian Formation that did not form a clade with any extant lineage[2]. Calibration 6 was placed on the stem node of *Bolitogyrus* Chevrolat (crown node of Cyrtoquediina) based on specimen #PAL591468 (NMNH, Table S1) from the Early Eocene Green River Formation, that can be assigned to the *Bolitogyrus* lineage based on evidence outlined in the main article. Calibration 7 was placed on a node leading to the stem of Philonthina based on *Philonthus marcidulus* Scudder from the Late Eocene Florissant formation[17]. Although most of Scudder's taxonomic placements of staphylinid fossils are erroneous (ranging from wrong genus to wrong subfamily) (Brunke and Solodovnikov, *pers. obs.*), several fossils described by him as species of *Philonthus* can at least be identified to the subtribe Philonthina, and *P. marcidulus* is among the most confidently placed based on a combination of the wide mesocoxal cavities and the posterior transverse basal line on the abdominal tergites[18]. Calibration 8 was placed on the node representing the most recent common ancestor shared between *Platydracus* Thomson*, Dinothenarus* Thomson and *Tasgius* Stephens (all Staphylinina), based on *Platydracus breviantennatus* Cai and Newton from the Late Eocene Florissant formation[19]. Calibration 9 was placed on the node representing the crown of the '*Tympanophorus* lineage' (Anisolinina) *sensu* Schillhammer (2004) based on *Tympanophorus greenwalti* Chatzimanolis, Brunke and Schillhammer from the Middle Eocene Kishenehn Basin[20] (NMNH). A slightly older (undescribed) fossil from the Early Eocene Green River formation (51 Mya) may also represent the *Tympanophorus*-lineage but this specimen is far less preserved[20].

Supplementary Results and Discussion

Possible Laurasian origins for Staphylinini, Arrowinini and Paederinae

Based on our divergence estimates, stem group Staphylinini (151.3, 138.7–166.0), Arrowinini (151.3, 138.7–166.0) and Paederinae (148.1, 135.7–162.6) originated in the Latest Jurassic, either well after (median ages) Gondwana and Laurasia had severed their last connections or soon after (limits of 95% HPD) (Fig. 4). These are unexpectedly young ages given the co-occurrence of dominantly northern or southern hemisphere lineages in these groups. Based on the paleogeological reconstructions[11] some island chains may have allowed faunistic exchange during 160-165 Mya but these dates are barely included in the 95% HPDs and would imply an extremely short period of stem evolution before the crown northern and southern hemisphere lineages became isolated. Indeed, our data so strongly influenced these divergence estimates toward younger stem ages for the above groups that the posterior estimates did not include ages that were given highest prior probability (*e.g.*, 170 Mya) in Analysis 1. Vertebrate fossil evidence suggests that, although Laurasia and Gondwana were separated by the Tethys Ocean at ~165 Mya, some Laurasian lineages have migrated from Europe to Africa and South America over a 'Eurogondwannan' connection while the latter two were still connected during the beginning of the Early Cretaceous (145–130 Mya)[21,22]. Thus, stem Staphylinini, Arrowinini and Paederinae may have originated in Laurasia or Gondwana, dispersed via this connection and, attained worldwide distributions that could be confused for classic distributions of Pangaen origin. The dominance of the north temperate clade of Staphylinini in the fauna of the Indian subcontinent and in Madagascar lends further support to a Eurogondwanan scenario and an origin in Laurasia rather than Gondwana. India and Madagascar were originally part of Gondwana but they were already physically separated from Africa by as early as the end of the Middle Jurassic (160 Mya)[23]. Under a Eurogondwanan dispersal scenario, Staphylinini would not have reached India until its more recent collision with the Asian plate 55 million years ago[24]. Although the ages of Staphylinini, Arrowinini and Paederinae were outside of the main scope of this study, these are the first estimates from a fossil-calibrated analysis that is based on a robustly supported phylogeny and challenge numerous earlier notions of a Pangaean origin based on extant distributions[25]; they should be tested when additional fossil evidence becomes available for these lineages.

The origin of the 'northern hemisphere' clade

Our analyses support a crown-group origin of the northern hemisphere clade far younger (124.4, 112.5–138.1) than the final breakup of Pangaea (170–165 Mya[13]) (Fig. 4) and significantly pre-dating the Late Cretaceous rise of angiosperm-dominated tropical forests[26]. A middle Early Cretaceous origin is consistent with the markedly poor representation of this lineage in the southern hemisphere, especially the south temperate region[1]. Congruently, the southern hemisphere groups of Staphylinini, here forming a relatively well supported clade (PP = 0.91) sister to the northern hemisphere clade, are poorly represented in the northern hemisphere. This is consistent with an Early Cretaceous origin of the former clade on a Gondwanan landmass[25]. The 95% HPD for the crown group age of both clades do partly overlap with the availability of the Eurogondwanan dispersal corridor but their extant distributions argue for younger origins and limited Laurasia-Gondwana exchange.

Evolution of major northern hemisphere lineages

The initial diversification of the northern hemisphere clade into major lineages occurred during the Early Cretaceous (median ages 103.8–124.4) (Table S2). This is consistent with existing fossil evidence, as none of the Staphylinini from the Early Cretaceous Yixian formation of China (126–124.6 Mya) were attributable to extant lineages[2], though this may have been due to missing data. Although the extant genus *Quedius*

(Quediina) has been reported from this formation[27], we believe this taxon to be a synonym of a previously described species of *Cretoquedius*, a stem-lineage of Staphylinini[2]. The identity of this fossil and a reassessment of its morphology are outside of the scope of this paper and will be addressed in a future investigation. Some characters that could place the Yixian Staphylinini in the crown northern hemisphere clade (*i.e.*, a sinuate basal elytral ridge[18]) were not preserved on any of the fossils. The present age estimates suggest that these fossil taxa do indeed belong to the stem rather than crown group of this lineage.

The Cretaceous was a time of major ecological change or 'Terrestrial Revolution' (KTR)[28] where Angiosperm plant communities gained dominance over Gymnosperms, possibly resulting in accelerated co-diversification with herbivorous insects, including butterflies[29], ants[30] and beetles[31]. Arid environments were rapidly becoming replaced by mesic ones during this period and based on our divergence estimates (Table S2), the major lineages of the northern hemisphere clade originated in wet, forested habitats, overlapping with the KTR (125–80 Mya[28]). We propose that the rise of ever-wet, equable climate forests[26] had an opposite effect on mesophilous Staphylinini diversification compared to that of tenebrionid beetles that generally originated under and are adapted to arid conditions, and experienced high levels of extinction during the KTR[14]. Although the major lineages of the northern hemisphere clade appear to have originated under similar conditions, they later diversified under, in some cases substantially, different paleoclimatic circumstances. For example, crown group Cyrtoquediina originated at or near (66.0 Mya) the Paleocene-Eocene hyperthermal (~65.5 Mya[32]), while crown group Quediina originated in the Middle Eocene (46.6 Mya) when the 'greenhouse' climate was transforming into an 'icehouse' climate[33]. Modern cyrtoquediines are generally thermophilic and associated with low seasonality[1], while quediines (*sensu*[1]) are obviously well-adapted to seasonal environments as they form dominant faunal elements in temperate and boreal forest litter communities[34]. Although an analysis of diversification rates was outside of the scope of the present study due to the inequalities in the depth and evenness of lineage sampling of our dataset[35], the diversification of Staphylinini was evidently very complex and warrants future study.

Conclusions

The unexpectedly young age of tribe Staphylinini challenges previous hypotheses about classical Gondwanan-Laurasian vicariance[25] as the cause of its main divergence into predominantly northern or southern hemisphere clades. Instead, we propose that Staphylinini originated on Laurasia during the Jurassic and dispersed to Gondwana through the 'Eurogondwanan' connection sometime in the earliest Early Cretaceous. This hypothesis brings a new framework within which to explore other biogeographic questions concerning this diverse and ancient lineage.

Supplementary references

1. Brunke, A. J., Chatzimanolis, S., Schillhammer, H. & Solodovnikov, A. Early evolution of the hyperdiverse rove beetle tribe Staphylinini (Coleoptera: Staphylinidae: Staphylininae) and a revision of its higher classification. *Cladistics* **32**, 427-451 (2016).
2. Solodovnikov, A., Yue, Y., Tarasov, S. & Ren, D. Extinct and extant rove beetles meet in the matrix: Early Cretaceous fossils shed light on the evolution of a hyperdiverse insect lineage (Coleoptera: Staphylinidae: Staphylininae). *Cladistics* **29**, 360-403 (2013).
3. McKenna, D. D. *et al.* Phylogeny and evolution of Staphyliniformia and Scarabaeiformia: forest litter as a stepping stone for diversification of nonphytophagous beetles. *Systematic Entomology* **40**, 35-60 (2015).
4. Drummond, A., Suchard, M., Xie, D. & Rambaut, A. Bayesian phylogenetics with BEAUti and the BEAST 1.7 *Molecular Biology and Evolution* **29**, 1969-1973 (2012).
5. Tracer v1.6, available from http://beast.bio.ed.ac.uk/Tracer (2014).
6. Ho, S. & Phillips, M. J. Accounting for Calibration Uncertainty in Phylogenetic Estimation of Evolutionary Divergence Times. *Systematic Biology* **58**, 367-380 (2009).

7. Parham, J. F. *et al.* Best Practices for Justifying Fossil Calibrations. *Systematic Biology* **61**, 346-359, doi:10.1093/sysbio/syr107 (2012).
8. Parker, J. & Grimaldi, D. A. Specialized myrmecophily at the ecological dawn of modern ants. *Current Biology* **24**, 2428-2434 (2014).
9. Schomann, A. S. *Hyperomma of New Zealand (Coleoptera: Staphylinidae: Paederinae). Systematics, phylogeny, historical biogeography* PhD. thesis, University of Copenhagen, (2014).
10. Ross, M. I. & Scotese, C. R. A hierarchical tectonic model of the Gulf of Mexico and Caribbean region. *Tectonophysics* **155**, 139-168 (1988).
11. Scotese, C. R. *Middle Jurassic paleographic map*. 295 (Geological Society of America Special Paper 288, 1994).
12. Dercourt, J., Ricou, L. E. & Vrielynck, B. *Atlas Tethys paleoenvironmental maps*. (Gauthier-Villars, 1993).
13. Bartolini, A. & Larson, R. L. Pacific microplate and the Pangea supercontinent in the Early to Middle Jurassic. *Geology* **29**, 735-738 (2001).
14. Kergoat, G. J. *et al.* Cretaceous environmental changes led to high extinction rates in a hyperdiverse beetle family. *BMC Evolutionary Biology* **14**, 1-13 (2014).
15. Chatzimanolis, S., Grimaldi, D. A., Engel, M. S. & Fraser, N. C. *Leehermania prorova*, the earliest staphyliniform beetle, from the Late Triassic of Virginia (Coleoptera: Staphylinidae). *American Museum Novitates* **3761**, 1-28 (2012).
16. Pasnik, G. & Kubisz, D. A new genus and new species of Staphylinidae (Coleoptera) from Baltic amber. *European Journal of Entomology* **99**, 353-361 (2002).
17. Scudder, G. Adephagous and clavicorn Coleoptera from the Tertiary deposits at Florissant, Colorado with descriptions of a few other forms and a systematic list of the non-rhynchophorus Tertiary Coleoptera of North America. . *Monographs of the United States Geological Survey* **40**, 1-148 (1900).
18. Brunke, A. & Solodovnikov, A. *Alesiella* gen.n. and a newly discovered relict lineage of Staphylinini (Coleoptera: Staphylinidae). *Systematic Entomology* **38**, 689-707 (2013)
19. Cai, C.-Y., Newton, A. F., Huang, D.-Y. & Tang, L. A new species of *Platydracus* Thomson, 1858 (Coleoptera, Staphylinidae, Staphylininae) from the upper Eocene Florissant beds, Colorado, USA. *Palaeoworld* **23**, 321-326 (2014).
20. Brunke, A., Chatzimanolis, S. & Schillhammer, H. The first fossil rove beetle from the middle Eocene Kishenehn Formation (North America) provides evidence for ancient Eocene relicts within the hyperdiverse Staphylinini (Coleoptera: Staphylinidae: Staphylininae). *Journal of Systematic Paleontology*, 1-11, doi: http://dx.doi.org/10.1080/14772019.2016.1266402 (2017).
21. Gheerbrant, E. & Rage, J.-C. Paleobiogeography of Africa: How distinct from Gondwana and Laurasia? *Palaeogeography, Palaeoclimatology, Palaeoecology* **241**, 224-246 (2006).
22. Ezcurra, M. D. & Agnolín, F. L. A New Global Palaeobiogeographical Model for the Late Mesozoic and Early Tertiary. *Systematic Biology* **61**, 553-566, doi:10.1093/sysbio/syr115 (2012).
23. McLoughlin, S. The breakup history of Gondwana and its impact on pre-Cenozoic floristic provincialism. *Australian Journal of Botany* **49**, 271-300 (2001).
24. Beck, R. A. *et al.* Stratigraphic evidence for an early collision between northwest India and Asia. *Nature* **373**, 55-58 (1995).
25. Solodovnikov, A. & Schomann, A. Revised systematics and biogeography of 'Quediina' of sub-Saharan Africa: new phylogenetic insights into the rove beetle tribe Staphylinini (Coleoptera: Staphylinidae). *Systematic Entomology* **34**, 443-466 (2009).
26. Morley, R. J. *Cretaceous and Tertiary Climate Change and the Past Distribution of Megathermal Rainforests.*, 1-31 (Praxis Publishing, 2007).
27. Cai, C.-Y. & Huang, D.-Y. A new species of small-eyed *Quedius* (Coleoptera: Staphylinidae: Staphylininae) from the Early Cretaceous of China. *Cretaceous Research* **44**, 54-57 (2013).
28. Lloyd, G. T. *et al.* Dinosaurs and the Cretaceous Terrestrial Revolution. *Proceedings of the Royal Society of London B: Biological Sciences* **275**, 2483-2490 (2008).
29. Wahlberg, N., Wheat, C. & Peña, C. Timing and patterns in the taxonomic diversification of Lepidoptera (butterflies and moths). *PLoS One* **8**, e80875 (2013).
30. Moreau, C., Bell, C., Vila, R., Archibald, S. B. & Pierce, N. Phylogeny of the ants: diversification in the age of angiosperms. *Science* **312**, 101-104 (2006).
31. McKenna, D. D., Sequeira, A. S., Marvaldi, A. E. & Farrell, B. D. Temporal lags and overlap in the diversification of weevils and flowering plants. *Proceedings of the National Academy of Sciences* **106**, 7083-7088, doi:10.1073/pnas.0810618106 (2009).
32. Zachos, J. C., Dickens, G. R. & Zeebe, R. E. An early Cenozoic perspective on greenhouse warming and carbon-cycle dynamics. *Nature* **451**, 279-283 (2008).
33. Zachos, J., Pagani, M., Sloan, L., Thomas, E. & Billups, K. Trends, rhythms, and aberrations in global climate 65 Ma to present. *Science* **292**, 686-693 (2001)
34. Smetana, A. Revision of the tribe Quediini of North America north of Mexico (Coleoptera: Staphylinidae). *Memoirs of the Entomological Society of Canada* **No. 79**, 1-303 (1971).
35. Renner, S. & Cusimano, N. Slowdowns in diversification rates from real phylogenies may be not real. *Systematic Biology* **59**, 458-464 (2010).
36. Grimaldi, D. A. & Engel, M. S. *Evolution of the Insects*. 1-755 (Cambridge University Press, 2005).
37. Perkovsky, E. E., Rasnitsyn, A. P., Vlaskin, A. P. & Taraschuk, M. V. A comparative analysis of the Baltic and Rovno amber arthropod faunas: representative samples. *African Invertebrates* **48**, 229-245 (2007).

38. Swisher, C. C. *et al.* Further support for a Cretaceous age for the feathered-dinosaur beds of Liaoning,China: New 40Ar/39Ar dating of the Yixian and Tuchengzi Formations. *Chinese Science Bulletin* **47**, 135-138 (2002).
39. Smith, M. E., Carroll, A. R. & Singer, B. S. Synoptic reconstruction of a major ancient lake system: Eocene Green River Formation, western United States. *Geological Survey of America Bulletin* **120**, 54-84 (2008).
40. Evanoff, E., McIntosh, W. C. & Murphey, P. C. in *Fossil Flora and Stratigraphy of the Florissant Formation, Colorado* Vol. 4 (eds E. Evanoff, K.M. Gregory-Wodzicki, & K.R. Johnson) 1-16 (Denver Museum of Nature & Science, 2001).
41. Constenius, K. N. Late Paleogene extensional collapse of the Cordilleran foreland fold and thrust belt. *Geological Survey of America Bulletin* **108**, 20-39 (1996).

Supplementary Figure

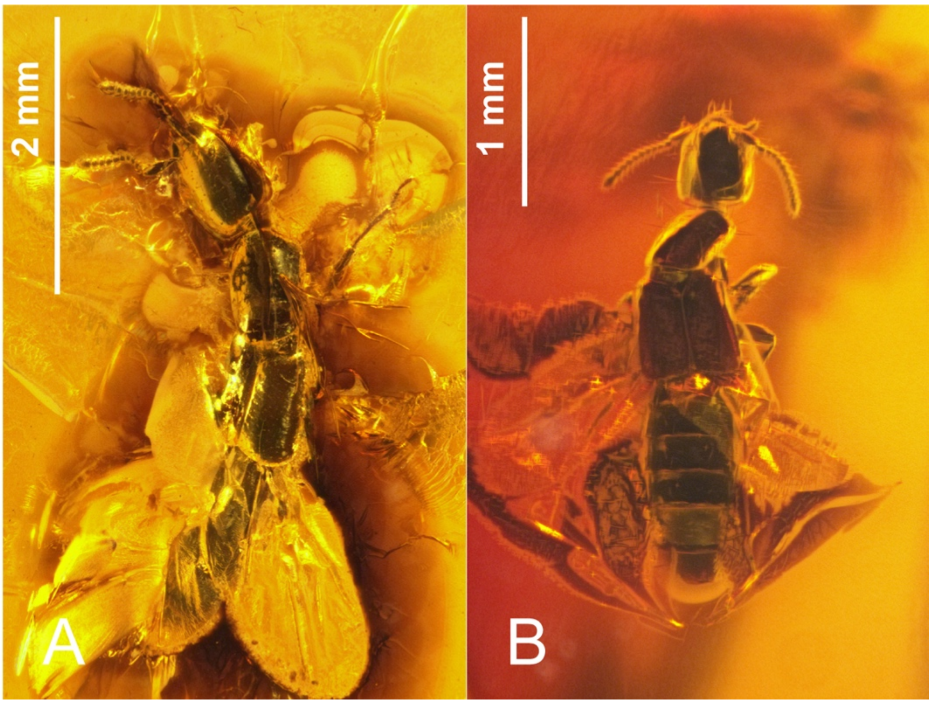

Figure S1. Undescribed fossil Xantholinini (Staphylinidae: Staphylininae): A) Xantholinini Genus A (Baltic Amber, 11A-5009, SIZK); B) Xantholinini Genus B (Baltic Amber, K329, SIZK).

Table S1. Node calibrations, geological ages and parameters for fossil prior distributions used in Analysis 1. The second analysis involved the same parameters but with uniform priors for node calibrations.

Calibration Node in Fig. 4	Taxon	Specimen #	Deposit and Age (Mya)	Prior distribution (Mya)
1. Root	*Leehermania prorova*	734a,b (VMNH)	Cow Formation, ~220[36]	Uniform = 220–0
2. Staphylininae: Xantholinini (crown)	Undescribed Xantholinini	11A-5009, K329 (SIZK)	Baltic Amber, 33.9-38.0[37]	Lognormal: offset = 33.9; mean = 20; SD = 0.5
3. Paederinae (stem)	*Mesostaphylinus antiquus*	LB2008783 (CNUB)	Yixian Formation, 126–124.6[38]	Lognormal: offset = 124; mean = 30; SD = 0.5
4. Paederinae: Lathrobiina (stem)	*Lathrobium ambricum*	1/1531/187/00 (ISEA)	Baltic Amber, 33.9-38.0[37]	Lognormal: offset = 33.9; mean = 20; SD = 0.5
5. Staphylinini (stem)	*Cretoquedius distinctus*	LB2008673P/C (CNUB)	Yixian Formation, 126.1–124.6[38]	Lognormal: offset = 124; mean = 30; SD = 0.5
6. *Bolitogyrus* (stem)	Undescribed *Bolitogyrus*	PAL591468 (NMNH)	Piceance Creek, Green River, 51.24 ± 0.52[39]	Lognormal: offset = 51; mean = 20; SD = 0.5
7. Philonthina (stem)	*Philonthus marcidulus*	1529, 1532 (MCZ)	Florissant, 34.07 ± 0.1[40]	Lognormal: offset = 34; mean = 20; SD = 0.5
8. *Platydracus* (stem)	*Platydracus breviantennatus*	35006 (YPM)	Florissant, 34.07 ± 0.1[40]	Lognormal: offset = 34; mean = 20; SD = 0.5
9. Tympanophorus lineage (stem)	*Tympanophorus greenwalti*	595140 (NMNH)	Kishenehn Basin, 46.2–43.5[41]	Lognormal: offset = 43.5; mean = 20; SD = 0.5

Table S2. Median age estimates and their 95% highest probability density (HPD) for Paederinae, Arrowinini (Staphylininae) and lineages of Staphylinini (Staphylininae). NHC – ‘northern hemisphere clade’.

**Lineage**	**Node**	**Analysis 1**		**Analysis 2**	
		Median	95% HPD	Median	95% HPD
Paederinae	Stem	148.1	(135.7–162.6)	145.1	(124.0–174.9)
	Crown	126.1	(108.0–143.2)	124.7	(101.8–155.8)
Arrowinini	Stem	151.3	(138.7–166.0)	149.1	(124.6–178.6)
	Crown	25.6	(15.8–37.2)	24.7	(16.4–35.5)
Staphylinini	Stem	151.3	(138.7–166.0)	149.1	(124.6-178.6)
	Crown	136.8	(123.6–151.1)	139.6	(117.5-169.7)
NHC	Stem	136.8	(123.6–151.1)	130.4	(108.7-158.2)
	Crown	124.4	(112.5–138.1)	120.5	(100.8-146.7)
Cyrtoquediina	Stem	124.4	(112.5–138.1)	120.5	(100.8-146.7)
	Crown	66.0	(57.1–77.2)	64.5	(51.1-81.8)
Bolitogyrus	Stem	66.0	(57.1–77.2)	64.5	(51.1-81.8)
	Crown	47.9	(38.6–59.4)	46.5	(34.0-61.0)
Acylophorina	Stem	113.8	(101.9–127.3)	110.7	(91.4-134.6)
	Crown	107.6	(94.4–120.6)	104.6	(85.6-127.8)
Erichsoniina	Stem	113.8	(101.9–127.3)	110.7	(91.4-134.6)
	Crown*	-	-	-	-
Indoquediina	Stem	109.6	(98.7–122.5)	107.4	(89.6-131.2)
	Crown	25.6	(17.3–34.1)	25.5	(16.7-35.7)
Quediina	Stem	103.8	(92.6–115.4)	101.8	(84.3-123.8)
	Crown	46.6	(39.6–54.7)	46.6	(37.3-59.1)
Staphylinini propria	Stem	103.8	(92.6–115.4)	101.8	(84.3-123.8)
	Crown	88.0	(78.7–98.4)	86.9	(71.6-108.1)
Xanthopygina	Stem	75.8	(64.5–87.4)	76.7	(61.6-95.3)
	Crown	49.7	(40.2–59.6)	50.2	(38.5-63.4)
Philonthina	Stem	81.0	(70.7–92.1)	79.1	(64.5-97.2)
	Crown	72.5	(62.5–83.6)	69.8	(57.3-87.2)
Staphylinina	Stem	73.9	(65.3–82.8)	73.5	(60.9-91.5)
	Crown	63.8	(54.9–73.3)	63.5	(49.8-79.0)
Anisolinina	Stem	73.9	(65.3–82.8)	73.5	(60.9-91.5)
	Crown	69.9	(61.7–78.8)	69.9	(57.1-86.9)

Table S3. Specimens and GenBank accession numbers for taxa used in phylogenetic analyses. Novel GenBank accessions are in bold and missing data are indicated with a dash (-). Outgroup taxa marked with '1' were composites of sequences from two congeners.

		Genbank accession numbers					
Taxon	Collection, Specimen #	28S	ArgK	CADA+CADC	CO1	TP	Wg
**Pseudopsinae**							
Pseudopsis arrowi Bernhauer 1939[1]	ZMUC, PSEarr01	/	KR259685	KR259797	KR259750	KR259744	-
Pseudopsis montoraria Herman 1975[1]	DDM0328	KJ845064	/	KJ845235 (A)	/	/	-
**Paederinae**							
Astenus sp. (U.S.A.)	UTCI, SC-0103	GU377318	KF178775	KF178807	GU377362	GU377412	GU377463
Hyperomma sp. 1[1] (Australia)	ZMUC, HYPnsw02	/	KR259669	KR259788 (C)	KR259767	KR259736	KR259704
Hyperomma sp. 2[1] (Australia)	DDM1149	KJ844900	/	KJ845300 (A)	/	/	/
Pseudolathra sp. (Laos)	ZMUC, PAE01	GU377340	KF178776	KF178808	GU377386	GU377437	GU377488
**Staphylininae**							
**Arrowinini**							
Arrowinus minutus Solodovnikov and Newton, 2005	ZMUN, 5422	KR559837	KT021926	KT000244	KT021964	KT022021	KT022056
Arrowinus relictus Solodovnikov and Newton, 2005	ZMUC, ARRrel01	-	KT021936	KR259799	GU377361	GU377411	GU377462
**Maorothiini**							
Maorothius brouni (Steel, 1948)	ZMUC, 00046196	KR559854	KT021881	KT000199	KT021965	KT021988	KT022061
**Othiini**							
Atrecus punctiventris (Fall, 1901)	SBMNH, SC-0104	GU377321	KT021882	KT000259	GU377365	GU377415	GU377466
Othius punctulatus (Goeze, 1777)	ZMUC, OTHpun01	GU377339	KT021883	KT000200	GU377385	GU377436	GU377487
Othius sp. (Japan)	UTCI, SC-0191	KR559858	KT021884	KT000201	KT021975	KT021989	KT022058
**Platyprosopini**							
Platyprosopus sp. (South Africa)	ZMUC, PLsp01	-	KF178777	KF178809	GU377391	GU377442	GU377493
**Xantholinini**							
Neohypnus sp. (Costa Rica)	ZMUC, 00046194	KR559823	KT021923	KT000241	KT021962	KT022019	KT022066
Nudobius pugetanus Casey 1906	UTCI, SC-0105	GU377335	KT021880	KT000198	GU377381	GU377432	GU377483
Lithocharodes sp. (Costa Rica)	ZMUC, 00046198	KR559824	KT021879	KT000197	KT021958	KT021987	KT022059
Thyreocephalus annulatus (Fauvel, 1895)	ZMUC, 00046195	KR559822	KT021878	KT000196	KT021954	KT021986	KT022069
**Staphylinini**							
Incertae sedis							
Afroquedius sexpunctatus (Bernhauer, 1917)	ZMUN, 5387	KR559843	KT021919	KT000237	KT021972	KT022018	KT022060
Algon hollowayae Schillhammer, 2006	ZMUC, ALsp01	GU377314	KF178787	KF178818	GU377356	GU377406	GU377457
Algon nr. *oculatus* Cameron, 1932	ZMUC, RIsp01	KF178761	KF178788	KF178819	KF178716	KF178748	KF178732
Antimerus punctipennis Lea, 1906	ZMUC, 00046183	KR559853	KT021918	KT000236	KT021974	KT022017	KT022045
Philothalpus bilobus Chatzimanolis and Ashe, 2005	UTCI, SC-0086	KF178762	-	KF178820	KF178717	-	KF178733
Philothalpus falini Chatzimanolis and Ashe, 2005	UTCI, SC-0008	KF178763	KF178789	KF178821	KF178718	KF178749	KF178734

Table S3. Specimens and GenBank accession numbers for taxa used in phylogenetic analyses. Novel GenBank accessions are in bold and missing data are indicated with a dash (-). Outgroup taxa marked with '1' were composites of sequences from two congeners.

		Genbank accession numbers					
Taxon	Collection, Specimen #	28S	ArgK	CADA+CADC	CO1	TP	Wg
**Acylophorina**							
Acylophorus capensis Cameron 1945	ZMUN, 5329	KR559831	KT021908	KT000226	KT021983	KT022011	KT022067
Acylophorus sp. (Bolivia)	ZMUC, 00046187	-	KT021910	KT000228	GU377355	GU377405	GU377456
Anaquedius vernix (LeConte)	ZMUC, AQsp01	GU377316	KT021907	KT000225	GU377358	GU377408	GU377459
Anchocerus sp. (Laos)	ZMUC, ANsp01	-	KT021937	KT000254	GU377359	GU377409	GU377460
Hemiquedius ferox (LeConte 1878)	ZMUC, 00046184	KR559838	KT021915	KT000233	KT021963	KT022016	KT022039
Paratolmerus siamensis Rougemont 1991	ZMUC, 00046186	KR559839	KT021909	KT000227	KT021980	KT022012	KT022056
**Amblyopinina**							
Amblyopinus emarginatus Seevers, 1955	UTCI, SC-0106	-	KT021940	KT000257	GU377357	GU377407	GU377458
Heterothops sp. (Costa Rica)	ZMUC, 00046197	KR559825	KT021885	KT000202	KT021976	KT021990	KT022062
Quediocafius taieriensis (Broun, 1894)	ZMUC, QCsp01	-	KT021938	KT000255	KT021971	KT022024	KT022064
Undescribed genus (eastern Australia)	ZMUC, 00046208	KR559855	KT021886	KT000203	KT021970	-	KT022063
**Anisolinina**							
Anisolinus sp. (Laos)	ZMUC, Alsp04	KR559828	KT021912	KT000230	-	KT022014	KT022042
Hesperosoma pederseni Schillhammer, 2009	ZMUC, 00046200	-	KT021933	KT000251	KT021950	-	KT022041
Misantlius gebieni Bernhauer, 1942	ZMUC, 00046193	KR559827	KT021911	KT000229	KT021966	KT022013	KT022040
Pammegus cf. *ruficollis* Fauvel, 1895	ZMUC, 00046201	KR559829	KT021913	KT000231	KT021978	KT022015	KT022055
Tolmerinus sp. (Laos)	ZMUC, Alsp01	GU377317	KF178778	KF178810	GU377360	GU377410	GU377461
Tympanophorus sp. (Laos)	ZMUC, TYsp01	GU377351	KF178779	KF178811	GU377401	GU377452	GU377503
**Cyrtoquediina**							
Astrapaeus ulmi (Rossi, 1790)	ZMUC, 00046192	KR559848	KT021894	KT000211	KT021969	KT021998	KT022049
Bolitogyrus bullatus (Sharp, 1884)	ZMUC, 00046173	KR559833	KT021888	KT000205	KT021959	KT021993	KT022052
Bolitogyrus falini Brunke, 2014	ZMUC, 00046177	**MF621983**	**MF621990**	**MF621993**	**MF621987**	**MF621999**	**MF621996**
Bolitogyrus flavus Yuan et al., 2007	ZMUC, 00046176	KR559835	KT021890	KT000207	KT021961	KT021995	KT022054
Bolitogyrus salvini (Sharp, 1884)	ZMUC, 00046175	KR559834	KT021889	KT000206	KT021960	KT021994	KT022053
Bolitogyrus silex Brunke, 2014	ZMUC, 00046174	**MF621985**	**MF621991**	**MF621994**	**MF621988**	-	**MF621997**
Bolitogyrus vulneratus (Fauvel, 1878)	ZMUC, BOsp01	GU377323	KT021891	KT000208	GU377367	GU377417	GU377468
Bolitogyrus pederseni Brunke, 2017	ZMUC, 00046170	**MF621984**	**MF621989**	**MF621992**	**MF621986**	**MF621998**	**MF621995**
Cyrtoquedius cf. *labiatus* (Erichson 1840)	ZMUC, QUsp01	KR559856	KT021893	KT000210	KT021968	KT021997	KT022044
Cyrtoquedius cf. *verecundus* Sharp 1884	ZMUC, 00046178	KR559836	KT021892	KT000209	KT021967	KT021996	KT022043
**Erichsoniina**							
Erichsonius (*Erichsonius*) *nanus* (Horn, 1884)	ZMUN, 22664	KR559830	KT021887	KT000204	KT021984	KT021991	KT022050
Erichsonsius (*Sectophilonthus*) sp. (Laos)	ZMUC, ERsp01	KR559857	-	-	KT021985	KT021992	KT022051
**Indoquediina**							
Indoquedius bicornutus Zhao and Zhou, 2010	ZMUN, 11473	-	KT021930	KT000248	KT021956	KT022022	KT022046
Indoquedius nr. *juno* (Sharp, 1874)	ZMUC, INsp01	-	KT021931	KT000249	GU377376	GU377427	GU377478
Indoquedius sp. (Thailand)	ZMUN, 22779	-	KT021932	KT000250	KT021957	-	KT022047

Table S3. Specimens and GenBank accession numbers for taxa used in phylogenetic analyses. Novel GenBank accessions are in bold and missing data are indicated with a dash (-). Outgroup taxa marked with '1' were composites of sequences from two congeners.

		Genbank accession numbers					
Taxon	Collection, Specimen #	28S	ArgK	CADA+CADC	CO1	TP	Wg
**Quediina**							
Euryporus picipes (Paykull 1800)	ZMUC, 00046169	KR559847	-	KT000219	KT021953	KT022005	KT022029
Korgella caucasica (Gusarov and Koval, 2002)	ZMUN, 12957	-	-	-	KT021941	-	KT022026
Quedionuchus plagiatus (Canada)	ZMUC, 00046168	KR559840	KT021906	KT000224	KT021945	KT022010	KT022036
Quedius antipodes Sharp 1840	ZMUC, QUsp05	-	KT021939	KT000256	GU377393	GU377444	GU377495
Quedius lateroflavus Lea 1925	ZMUC, QUlat01	-	-	KT000258	KT021977	KT022025	KT022065
Quedius (*Distichalius*) *alticola* Smetana 1971	ZMUN, 22670	KR559846	KT021903	KT000221	KT021942	KT022007	KT022030
Quedius (*Distichalius*) *capucinus* (Gravenhorst 1806)	UTCI, SC-0289	KR559852	KT021904	KT000222	KT021943	KT022008	KT022031
Quedius (*Microsaurus*) *beesoni* Cameron 1932	ZMUN, 22672	KR559844	KT021899	KT000216	KT021955	KT022003	KT022028
Quedius (*Microsaurus*) *cruentatus* (Olivier 1795)	ZMUC, QUcru01	GU377347	KT021898	KT000215	GU377394	GU377445	GU377496
Quedius (*Microsaurus*) *peregrinus* (Gravenhorst 1806)	ZMUC, 00046179	KR559841	-	-	KT021944	KT022002	KT022027
Quedius (*Paraquedius*) *puncticeps* Horn 1878	ZMUC, 00046180	KR559851	KT021900	KT000217	KT021951	-	KT022034
Quedius (*Quedius*) *molochinus* (Gravenhorst 1806)	ZMUC, QUmol01	GU377348	KF178783	KF178814	GU377396	GU377447	GU377498
Quedius (*Quedius*) *simplicifrons* (Fairmaire 1861)	ZMUC, 00046190	KR559849	KT021902	KT000220	KT021946	KT022006	KT022035
Quedius (*Raphirus*) *picipes* (Mannerheim 1830)	ZMUC, QUpic01	-	KT021928	KT000246	GU377395	GU377446	GU377497
Quedius (*Raphirus*) *prostans* Horn 1878	ZMUN, 22671	KR559832	KT021896	KT000213	KT021948	KT022000	KT022032
Quedius (*Raphirus*) *riparius* Kellner 1843	ZMUN, 22673	KR559845	KT021905	KT000223	KT021952	KT022009	KT022038
Quedius (*Raphirus*) *seriatus* Horn 1878	ZMUC, 00046181	KR559850	KT021897	KT000214	KT021949	KT022001	KT022033
Quedius (*Raphirus*) *suturalis* Kiesenwetter 1845	ZMUC, QUsut01	-	KT021929	KT000247	GU377397	GU377448	GU377499
Quedius (*Velleius*) *dilatatus* (1787)	ZMUC, VELsp01	GU377352	KT021917	KT000235	GU377402	GU377453	GU377504
Quemetopon grandipenis (Zhu et al. 2006)	ZMUN, 11650	KR559842	KT021901	KT000218	KT021979	KT022004	KT022037
Queskallion dispersepunctatum (Scheerpeltz 1965)	ZMUN, 11597	KR559826	KT021895	KT000212	KT021947	KT021999	KT022048
**Philonthina**							
Cafius seminitens Horn, 1884	UTCI, SC-0002	-	KF178781	KF178812	GU377368	GU377418	GU377469
Chroaptomus centralis Chani-Posse, 2006	UTCI, SC-0179	-	KT021934	KT000252	KT021981	KT149214	KT149215
Hesperus laevigatus Fauvel 1895	ZMUC, HSsp01	GU377330	KT021914	KT000232	GU377374	GU377425	GU377476
Neobisnius occidentoides Frank, 1981	UTCI, SC-0082	-	KT021935	KT000253	GU377379	GU377430	GU377481
Philonthus splendens (Fabricius, 1793)	ZMUC, PHspl01	-	KF178782	KF178813	GU377387	GU377438	GU377489
**Staphylinina**							
Creophilus maxillosus Linnaeus, 1758	UTCI, SC-0235	KF178760	KF178784	KF178815	KF178714	KF178746	KF178730
Dinothenarus saphyrinus LeConte, 1861	UTCI, SC-0001	GU377324	-	KT000238	GU377369	GU377419	GU377470
Platydracus cinnamopterus Gravenhorst, 1802	UTCI, SC-0081	GU377342	KT021920	KT000239	GU377389	GU377440	GU377491
Tasgius pedator Gravenhorst, 1802	ZMUC, TAped01	GU377349	KT021921	KT000240	GU377399	GU377450	GU377501
Thinopinus pictus LeConte, 1852	UTCI, SC-0003	GU377350	KT021916	KT000234	GU377400	GU377451	GU377502
**Tanygnathinina**							
Atanygnathus acuminatus (Casey 1915)	UTCI, MSC-1454	GU377319	KF178786	KF178817	GU377363	GU377413	GU377464
Atanygnathus sp. (Laos)	ZMUC, ATsp01	GU377320	KT021924	KT000242	GU377364	GU377414	GU377465

Table S3. Specimens and GenBank accession numbers for taxa used in phylogenetic analyses. Novel GenBank accessions are in bold and missing data are indicated with a dash (-). Outgroup taxa marked with '1' were composites of sequences from two congeners.

		Genbank accession numbers					
Taxon	Collection, Specimen #	28S	ArgK	CADA+CADC	CO1	TP	Wg
**Xanthopygina**							
Glenus flohri Sharp 1884	UTCI, SC-0172	KF178765	KF178792	KF178825	KF178720	KF178750	KF178736
Isanopus sallaei Sharp 1884	UTCI, SC-0202	KF178766	KF178793	KF178826	KF178721	KF178751	KF178737
Nordus fungicola Sharp 1884	UTCI, SC-0006	GU377334	KF178795	KF178828	GU377380	GU377431	GU377482
Plociopterus sp. (Costa Rica)	UTCI, SC-0197	KF178769	KF178799	KF178832	KF178724	KF178754	KF178740
Xanthopygus chapareanus Scheerpeltz 1969	UTCI, SC-0102	GU377353	KF178803	KF178835	GU377403	GU377454	GU377505
